# Modeling startle eyeblink electromyogram to assess fear learning

**DOI:** 10.1111/psyp.12775

**Published:** 2016-10-18

**Authors:** Saurabh Khemka, Athina Tzovara, Samuel Gerster, Boris B. Quednow, Dominik R. Bach

**Affiliations:** ^1^ Department of Psychiatry, Psychotherapy and PsychosomaticsUniversity of ZurichZurichSwitzerland; ^2^ Neuroscience Centre Zurich, University of ZurichZurichSwitzerland; ^3^ Wellcome Trust Centre for Neuroimaging, University College LondonLondonUK

**Keywords:** Fear‐potentiated startle, Fear conditioning, Electromyography, Psychophysiological model, Affective startle modulation

## Abstract

Pavlovian fear conditioning is widely used as a laboratory model of associative learning in human and nonhuman species. In this model, an organism is trained to predict an aversive unconditioned stimulus from initially neutral events (conditioned stimuli, CS). In humans, fear memory is typically measured via conditioned autonomic responses or fear‐potentiated startle. For the latter, various analysis approaches have been developed, but a systematic comparison of competing methodologies is lacking. Here, we investigate the suitability of a model‐based approach to startle eyeblink analysis for assessment of fear memory, and compare this to extant analysis strategies. First, we build a psychophysiological model (PsPM) on a generic startle response. Then, we optimize and validate this PsPM on three independent fear‐conditioning data sets. We demonstrate that our model can robustly distinguish aversive (CS+) from nonaversive stimuli (CS‐, i.e., has high predictive validity). Importantly, our model‐based approach captures fear‐potentiated startle during fear retention as well as fear acquisition. Our results establish a PsPM‐based approach to assessment of fear‐potentiated startle, and qualify previous peak‐scoring methods. Our proposed model represents a generic startle response and can potentially be used beyond fear conditioning, for example, to quantify affective startle modulation or prepulse inhibition of the acoustic startle response.

Predicting threat from environmental events is a fundamental ability of humans and many nonhuman species, and engages species‐specific defensive responses to facilitate survival. To investigate such fear memory, classical (Pavlovian) fear conditioning paradigms are commonly used, in which an initially neutral conditioned stimulus (CS) predicts an upcoming aversive unconditioned stimulus (US). Besides addressing a basic associative learning mechanism, such paradigms are also thought to model psychiatric conditions in humans such as posttraumatic stress disorder (PTSD) and other anxiety disorders (Lissek et al., 2005; VanElzakker, Dahlgren, Davis, Dubois, & Shin, 2014). Consequently, fear conditioning is used to develop interventions for the prevention or erasure of pathological fear (Carmichael & Lockhart, 2012; Grillon, Cordova, Morgan, Charney, & Davis, 2004; Reist, Duffy, Fujimoto, & Cahill, 2001; Schiller et al., 2010). Such investigations require an ability to detect subtle differences in fear memory strength. In rodent species, fear conditioning with unescapable foot shock results in freezing behavior that is easy to quantify (LeDoux, 1998). In contrast, humans do not exhibit overt freezing. Instead, human fear memory is often assessed via activity of the autonomic nervous system as measured with skin conductance response (SCR; Bach, Daunizeau, Friston, & Dolan, 2010; Staib, Castegnetti, & Bach, 2015), heart period response (HPR; Castegnetti et al., 2016), or pupil size (Kluge et al., 2011; Korn, Staib, Tzovara, Castegnetti, & Bach, 2016; Reinhard, Lachnit, & König, 2006). Yet, SCR are susceptible to internal emotional, cognitive, and motor processes unrelated to fear memory and typically require long intertrial intervals (ITIs) because of slowness of the peripheral signal (Boucsein, 2012; Hamm & Vaitl, 1996). Similarly, HPR may reflect motor preparation independent of stimulus valence (Hamm & Vaitl, 1996).

In light of these limitations, an interesting approach for assessing fear memory is to measure an increase of the innate startle response, a phenomenon termed fear‐potentiated startle (Blumenthal et al., 2005; J. S. Brown, Kalish, & Farber, 1951; Grillon, Ameli, Woods, Merikangas, & Davis, 1991; VanElzakker et al., 2014; Vanman, Boehmelt, Dawson, & Schell, 1996). In contrast to autonomic indices, this measure is less prone to nonspecific arousal, specifically measures aversive (rather than appetitive) learning (Lipp, Sheridan, & Siddle, 1994), and allows fear memory quantification across the life span (Grillon & Baas, 2003). Critically, it also affords direct comparison to many nonhuman species (Ameli, Ip, & Grillon, 2001; Grillon & Baas, 2003). Thus, unlike freezing or various autonomic indices, it comprises a truly translational measure and can be assessed even in simple organisms such as *Aplysia* (Carew, Walters, & Kandel, 1981).

In general, the startle reflex is a fast defensive response to an unexpected intense auditory, visual, or haptic stimulus, and appears to be aimed at protecting an organism from an imminent blow to the head (Yeomans, Li, Scott, & Frankland, 2002). It results in a postural change and, particularly easy to measure, an eyeblink response (Lang, Bradley, & Cuthbert, 1997). While this response pattern is rather stereotypical, its vigor appears adapted to trade off costs and benefits (Bach, 2015), leading to a higher startle magnitude when an attack is evaluated to be more likely such as during the time point of an expected US. To quantify fear learning in humans, one usually uses electromyography (EMG) to record the response of the musculus orbicularis oculi to loud tones with fast rise times, presented at anticipated US onset (Blumenthal et al., 2005), which we term here *startle eyeblink response* (SEBR).

To quantify SEBR magnitude, previous studies have used measures of area under the curve, peak amplitude, or peak latency of a preprocessed EMG (Blumenthal et al., 2005). Crucially, however, there is a lack of consensus in selecting the preprocessing steps, most reliable target measures, and the time window to search for a response (Blumenthal et al., 2005; Grillon et al., 1991). Thus, it often appears that analysis settings depend on the laboratory or even on experiment‐specific considerations, rather than on systematic investigations of robustness and sensitivity to detect differences between SEBR to CS+ and CS‐.

The goal of this study was, therefore, to fill this lacuna and to systematically investigate the sensitivity of different strategies for SEBR analysis. Importantly, each analysis scheme makes (implicit) assumptions on how the SEBR is generated, but uses only a limited number of data features to quantify SEBR, such as peak amplitude. We have previously demonstrated for SCR, HPR, respiratory measures, and pupil size responses that such implicit assumptions can be made explicit in a psychophysiological model (PsPM). This model specifies, in mathematical form, the expected shape of the response (Bach et al., 2010; Bach, Flandin, Friston, & Dolan, 2009; Bach, Gerster, Tzovara, & Castegnetti, 2016; Korn & Bach 2016; Paulus, Castegnetti, & Bach, 2016). The shared variance between expected response with unit amplitude and actual data, assessed, for example, in a regression model, can then be used to quantify response magnitude. This approach makes use of the entire data time series and theoretically affords more robust fear memory assessment—something we have shown empirically for SCR (Bach et al., 2010; Staib et al., 2015), HPR (Castegnetti et al., 2016), and pupil size (Korn et al., 2016). Here, we seek to create a model for SEBR in the existing PsPM framework.

To this end, we assume that SEBR is the output of a linear time invariant system, which is characterized by its impulse response function. We investigate whether SEBR has a stereotypical shape and timing, and create a PsPM for SEBR. In a second step, we used an independent fear retention data set in which CS+/CS‐ learning was well established, to examine whether the SEBR amplitude, estimated by inversion of this PsPM, differentiates between CS+ and a CS‐ (which we term *predictive validity*; Bach & Friston, 2013). In line with previous SCR approaches (Bach, Friston, & Dolan, 2013), the method was then optimized with respect to predictive validity, and validated on an independent fear retention and an additional fear acquisition data set. At the same time, we compare the predictive validity of our model‐based approach to four established SEBR analysis methods using Bayesian model comparison as previously established (Bach, 2014). To put these methods into psychophysiological context, we finally compare SEBR with the predictive validity afforded by SCR and HPR.

## Method

### Design and Participants

Experiment 1 was designed for the development of a quantitative SEBR model, such that we measured SEBR in the absence of any other manipulation. Experiment 2 used a fear‐potentiated startle design to determine the optimal model structure and preprocessing steps for inferring fear retention under extinction from SEBR. Experiment 3 served as independent validation of results from Experiment 2. In Experiment 4, we sought to demonstrate that our model‐based approach can be used to quantify fear acquisition. Experiment 2–4 used visual stimuli as CS, and an unpleasant electric stimulation as US.

Four independent samples were recruited from the student and general population in Zurich: 20 participants (13 females, age range 19–33 years, mean age ± *SD*: 22.84 ± 3.35 years) for Experiment 1, 23 participants (16 females, 19–33 years, 25.6 ± 4.22 years) for Experiment 2, 35 participants (23 females, 18–31 years, 23.3 ± 1 years) for Experiment 3, and 18 participants (nine females, 19–33 years, 23.12 ± 3.3 years) for Experiment 4. One participant in Experiment 1 and one in Experiment 3 did not complete the experiment and were excluded. Three participants from Experiment 2, four from Experiment 3, and three from Experiment 4 were excluded because of EMG, SCR, or US electrode detachment during the experiment. Experiment 3 included a startle group (15 participants), and a no‐startle group (15 participants). We sought to demonstrate the robustness of our method, and therefore did not exclude any nonresponders (no/low EMG response to startle stimuli) from data analysis. All participants gave written informed consent. The experiment was conducted in accordance with the Declaration of Helsinki, and its study protocol, including the form of taking consent, was approved by the governmental ethics committee (Kantonale Ethikkomission Zurich).

### Task Procedure

In Experiment 1 (data set code: SMD), we presented 25 acoustic startle probes randomly with an ITI of 7 to 11 s (mean 9 s) while participants fixated a white cross on a black computer screen.

Experiment 2 (data set code: DoxMemP) was designed to measure fear retention under extinction. During an acquisition phase, participants were presented with a 4‐s CS+ or CS‐ (red or blue screen background) in which 50% of CS+ trials coterminated with a 0.5‐s electric stimulation as US. The color/CS association was balanced across participants, and participants were not instructed about the identity of CS+ and CS‐. In total, 180 trials at an ITI of 7 to 11 s (mean 9 s) were presented during the acquisition phase. During an extinction session 1 week later, participants were presented with 20 CS+ and 20 CS‐ in randomized order, but without any US. On every extinction trial, a startle probe occurred at expected US onset (i.e., 3.5 s after CS onset). Fear memory expression decays under extinction such that the number of trials to analyze must strike a balance between excluding trials with decayed responses and minimizing measurement noise, which requires more trials. Previous studies using SEBR to assess fear extinction have typically reported a reduction in the strength of fear memories after 2–6 trials (Andreatta & Pauli, 2015; L. A. Brown, LeBeau, Chat, & Craske, 2016; Lindner et al., 2015). Here, we used the first 5 CS+ and 5 CS‐ trials of Experiment 2 and 3 for building and validating our model. We additionally include an exploratory post hoc analysis to investigate the performance of our model as a function of the number of included trials.

Experiment 3 (data set code: FR) was used to assess fear memory under extinction similar to Experiment 2 but with minor differences during the extinction phase. Fear retention was evaluated 1 day after acquisition. Six CS+/CS‐ were presented together with a startle probe 3.8 s after CS onset. In line with Experiment 2, we analyzed five CS+ and five CS‐ trials to differentiate CS+/CS‐. We included an additional group of participants in which no startle probes were presented, neither during retention nor during acquisition, such that we could analyze SCR and HPR during retention.

Experiment 4 (data set code: SS) investigated fear memory during acquisition with a design similar to the acquisition phase of Experiment 2. Eighty trials were presented with a random ITI of 7 to 11 s (mean 9 s). CS+ coterminated with the US on 25% of the trials. On 25% of the CS+ (always nonreinforced) and 25% of the CS‐ trials, a startle sound was presented at the anticipated US onset time (i.e., 3.5 s after of CS+ onset).

### Stimuli and Apparatus

In accordance with guidelines from Blumenthal (1988) and Blumenthal et al. (2005), white noise sounds of 50 ms length with < 2 ms onset ramp and ∼100 dB sound pressure were used as startle probes and delivered via headphones (Sennheiser HD 201, Germany), using the PC's in‐built sound card (Realtek high definition audio) and an external sound amplifier (K4102, Velleman, Belgium). Sound volume was determined offline using a white noise sound of 2 s duration and a sound level meter (SL‐200, Voltcraft, Germany). For Experiment 1–2, sound onset was controlled by recording the output of the sound card together with EMG, and all analyses relate to the measured startle sound onset. For Experiment 3–4, we ran the experiment scripts post hoc and recorded the output of the sound card together with event markers, for 300 and 120 sounds, respectively. We then corrected the event markers that were recorded together with the EMG by the minimum measured sound onset delay per experiment. This reflects a realistic scenario in many laboratories where only event markers but not startle sounds are measured together with EMG.

In Experiment 2–4, the US was a 500‐ms train of 250 square pulses with individual pulse width of 1 ms (Experiment 2 and 3) or 0.2 ms (Experiment 4). The US was delivered via a pin‐cathode/ring‐anode configuration attached on the participant's right forearm using a constant current stimulator (Digitimer DS7A, Digitimer Ltd, UK). US intensity was calibrated for each individual to a clearly uncomfortable level by adapting the current amplitudes in three phases before the start of the experiment. First, current was increased from 0.5 mA in steps of 0.5 mA to a level where the participant reported that the stimulus was clearly painful. Next, participants received 14 randomly selected stimulations below the pain threshold while the participant rated perceived intensity on a 0 (*no shock detected*) to 100 (*painful shock*) scale. Finally, the final intensity was set just below the reported pain threshold to a clearly unpleasant level (intensity mean ± *SD*: 2.82 ± 1.64 mA, 3.0 ± 1.50 mA, and 3.2 ± 1.44 mA for Experiment 2–4, respectively).

Eyeblink responses were measured via EMG activity of the periorbital region of the musculus orbicularis oculi using a pair of 4 mm Ag/AgCl cup electrodes. One of them was placed approximately 10 mm below the lower eyelid in line with the pupil in forward gaze and the other on the external canthus, at a distance of approximately 10 mm from the first (Blumenthal et al., 2005; Grillon et al., 1991). EMG signals were amplified using a Coulbourn high‐gain bioamplifier (V75‐04; Coulbourn Instruments, Whitehall, PA) with analogue band‐pass filter (13 Hz–1 kHz), an amplifier coupling of 1 Hz, and adjustable gain. The output signal was digitized at 1 kHz using a Dataq card (DI‐149, Dataq Inc., Akron, OH) and recorded with Windaq (Dataq Inc.) software for the entire duration of the experiment.

In the acquisition phase of Experiment 2–4, and in the no‐startle group in the retention phase of Experiment 3, we recorded skin conductance from the thenar/hypothenar of participants' nondominant hand, using 8 mm Ag/AgCl cup electrodes (EL258, Biopac Systems Inc., Goleta, CA) and 0.5% NaCl gel (GEL101, Biopac; Hygge & Hugdahl, 1985). Skin conductance signal was amplified with an SCR coupler/amplifier (V71‐23, Coulbourn Instruments). Further, we recorded electrocardiogram (ECG) through four 45 mm, pregelled Ag/AgCl adhesive electrodes, attached to the limbs. Prior to the experiment, the experimenter chose, for each participant, the lead (I, II, III) or augmented lead (aVR, aVL, aVF) configuration that provided the highest R spike. ECG was preamplified and 50 Hz notch‐filtered with a Colbourn isolated five‐lead amplifier (LabLinc V75‐11, Coulbourn Instruments).

### Data Preprocessing

All data were analyzed in MATLAB (version R2013b; MathWorks, Natick, MA) with PsPM 3.0 (http://pspm/sourceforge.net) and custom code that is available from the authors.

Continuous EMG data were initially filtered using a 4th order Butterworth band‐pass filter with cutoff frequency of 28 Hz and 250 Hz, following previous literature (Barker, Reeb‐Sutherland, & Fox, 2014). Mains noise was removed with a 50 Hz notch filter. Filtered continuous EMG signals were rectified and smoothed using a 4th order Butterworth low‐pass filter with a time constant of 3 ms corresponding to a cutoff frequency of 53.05 Hz (Blumenthal et al., 2005). In subsequent steps, we optimized initial preprocessing filter frequencies on data from Experiment 2.

Skin conductance data were filtered with a bidirectional Butterworth 1st order filter and cutoff frequencies of 0.0159 and 5 Hz, and downsampled to 10 Hz. They were analyzed with a nonlinear model (dynamic causal model, DCM) of the anticipatory SCR. This procedure infers activity of the sympathetic nervous system, given changes in the recorded SCR signal (Bach et al., 2010), and provides an estimate of sympathetic arousal on a trial‐by‐trial level (Bach et al., 2010; Staib et al., 2015). We used only those CS+ trials that were not paired with a US (CS+/US‐ trials) or startle sound, to avoid contamination by the electric stimulus or by motion artifacts. To put this method into context, we also computed a model‐free peak‐scoring measure of the anticipatory SCR as implemented in the PsPM function scr_peakscore (Boucsein, 2012). We defined a window of 1 to 4.5 s after CS onset, within which we searched for the onset of a SCR, similar to our previous work (Staib et al., 2015). It is recommended to only analyze responses with a minimum onset latency of 1 s (Boucsein, 2012), thus motivating the start of this window. The end of this window, 1 s after the anticipated US onset, ensures that SCR to the US omission are not taken into account. After finding the onset of a SCR, the actual SCR peak was identified within a second window, starting from 0.5 to 5 s after the SCR onset, in keeping with standard recommendations (Boucsein, 2012).

#### Preprocessing of HPR data

QRS complexes were identified semiautomatically in the ECG data through a modified offline version (Paulus et al., 2016) of the Pan and Tompkins (1985) real‐time QRS detection. Interbeat intervals were then linearly interpolated at 10 Hz to create a time series of equidistant time points (Paulus et al., 2016), which were band‐pass filtered with a bidirectional Butterworth filter (0.015–0.5 Hz). HPR were analyzed with a model‐based approach. We implemented a single‐component canonical response function (i.e., conserved across subjects) in a general linear convolution model, in which the average response amplitude is estimated as a free parameter (Castegnetti et al., 2016). Reconstructed HPR were used to identify the maximum signed deviation from baseline in response to CS+ and CS‐, within a window of 0 to 11 s poststimulus onset. These estimates were contrasted in order to quantify fear memory, similar to our previous work (Castegnetti et al., 2016).

### SEBR Model Specification

#### Linear time‐invariant systems

We assumed that the SEBR *y*(t) is the output of a linear time‐invariant (LTI) system with the defining properties linearity and time invariance. By linearity, input and output are linearly mapped so the responses to several inputs can be obtained by summing the responses to individual inputs. Time invariance means that the output does not explicitly depend on time. In principle, linearity ensures pure summation of two overlapping inputs, which may be unrealistic for startle response. However, because startle responses are not measured in quick succession, violations of this assumption bear little relevance for our model. We note that this could be relevant if one sought to apply the model to prepulse inhibition paradigms in which the prepulse itself can sometimes elicit startle responses (Blumenthal et al., 2005).

Mathematically, the output *y*(t) of a LTI system can be fully described by convolving input *x*(t) with the system's response function *h*(t) and can be written as

$$y\left(t\right)=x\left(t\right)*h\left(t\right)=\int_{0}^{\infty }x\left(t-\;\tau \right)\;h\left(\tau \right)\;d\tau$$

Here, we assume *x*(t) is an instantaneous (delta) input at startle sound onset. This implies that the SEBR is constant between trials. We sought to develop empirically *h*(t), the response function (RF) for SEBR.

#### Models

Data from Experiment 1 were used to construct the RF of the presumed LTI system. We extracted epochs of 500‐ms duration after the onset of each startle sound. Individual responses from all participants were entered into principal component analysis (PCA). The first principal component (PC) was approximated with a gamma distribution with shape parameter *k*, scale parameter *θ*, time onset *x_0_*, and amplitude *A*. The best‐fitting parameters for this gamma distribution were determined by minimizing the residual sum of squares using the Nelder‐Mead simplex direct search algorithm implemented in the MATLAB function *fminsearch* (Lagarias, Reeds, Wright, & Wright, 1998).

$$y^{\prime }=\frac{A}{\theta^{k}\Gamma (k)}\;{\left(x-x_{0}\right)}^{k-1}e^{-\frac{\left(x-x_{0}\right)}{\theta }},$$

**Figure 1:**
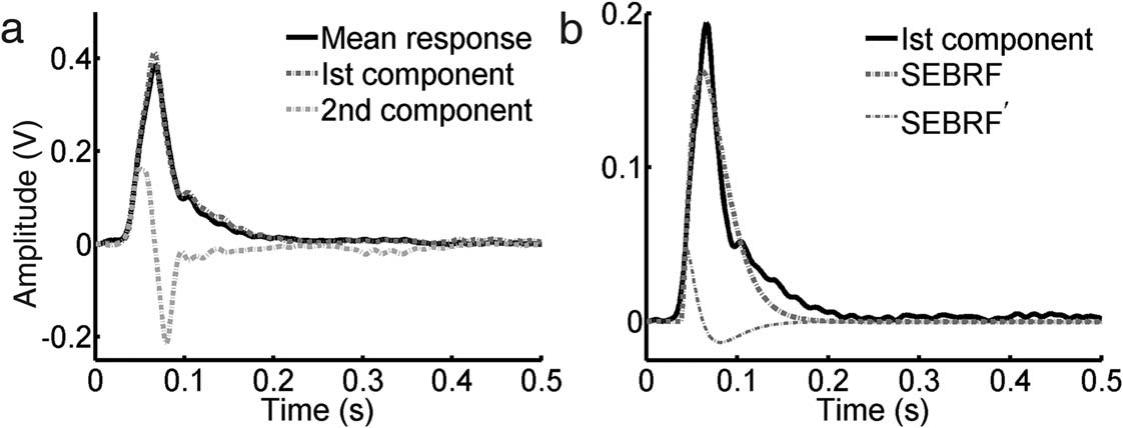
Startle eyeblink response (SEBR) for Experiment 1. a: SEBR (black line), averaged across all trials and participants, together with the first and second principal components (PCs). The second PC resembles the derivative of first PC. b: Fitted gamma response function and its time derivative (SEBRF and SEBRF'; M2) of the first PC.

We term this the canonical startle eyeblink response function (SEBRF) and formalize it as model M1 (Figure 1). The second PC resembled a time derivative of the first component. Rather than approximating the second component, we directly computed the time derivate of the gamma response function (SEBRF'), which was included together with the SEBRF into a model M2, analogous to previous models for SCR (Bach et al., 2009, 2013), HPR (Castegnetti et al., 2016; Paulus et al., 2016), respiration (Bach et al., 2016), and fMRI (Friston et al., 1998). Since the tail of the first PC component was not well fitted by M1 (Figure 1), we created a third model M3 that combined the SEBRF with a Gaussian function to model the response tail. Additional components of M2 and M3 were orthogonalized to the canonical SEBRF using the Gram‐Schmidt algorithm. The time derivative of the first component in model M2 can potentially account for small variations in the startle latency, as long as they are small compared to the overall SEBR duration. Larger variations may occur physiologically, or also because of variations in the precise startle sound onset, which is often not recorded online. To more explicitly account for such variation, we created model M4, which was equivalent to model M1 but with onset latency as a free parameter, to be estimated from the data.

#### General linear models

Under the assumption that SEBR are the output of an LTI system that receives an input with constant shape and latency but variable amplitude (M1–M3), we can harness general linear convolution modeling (GLM) to estimate the amplitude of the input. This GLM can be written as

Y=βX+ε,

where *X* is design matrix in which each column is obtained by convolving impulse functions at startle onset with each component of the RF. *Y* is the vector of observations (time series data), β is a vector of input amplitude parameters, and ϵ is the error that is assumed to be independent and identically distributed. The maximum‐likelihood amplitude estimates $\hat{\beta }$ are computed using the Moore‐Penrose pseudoinverse, implemented in MATLAB function *pinv*. We can then compute the estimates for different experimental conditions (CS+ and CS‐ mean startle amplitude in our case). In case of several basis functions, such as model M2 and M3, we reconstructed the estimated SEBR from the entire basis set and quantified startle response amplitude as the signed absolute variation from zero over a time window of 500 ms, analogous to previous approaches (Bach et al., 2013). GLMs were computed by collapsing all trials for each condition into one (M1) or two (M2/M3) regressors. In an additional approach M2ST, we accounted for trial‐by‐trial fluctuations in startle latency by modeling each trial with two unique regressors.

#### Dictionary‐matching algorithm

To invert model M4, startle latency had to be estimated, which obviates a GLM approach. We finessed this problem by using a dictionary‐matching algorithm. Each element in our dictionary specified a unit startle response described by model M1, at all potentially observable latencies given the discrete time resolution. We considered latencies between *x*_0_ − 0.02 s to *x*_0_ + 0.13 s for Experiment 2. For Experiment 3–4, we expanded this window by twice the standard deviation of the measured sound onset delay. The dictionary was then multiplied with the data, and the element that maximized the signed inner product with the data was entered as regressor into a GLM and used to estimate the SEBR amplitude for all regressors concurrently. We either specified one dictionary for all trials per condition in the entire experiment (M4) or one dictionary per trial (M4ST).

### Filter Optimization

Filter settings can have an impact on model performance (Bach et al., 2013; Staib et al., 2015). If the true response function is known, the optimal filter can be determined using the matched filter theorem. As this is not the case here, we take an empirical approach to filter optimization and use Bayesian model comparison to evaluate predictive validity under different filter parameters. We varied the high‐pass cutoff frequency between 10 to 90 Hz and the low‐pass cutoff frequency between 200 to 490 Hz in steps of 10 Hz. For each combination of high‐ and low‐pass cutoff frequencies, we recomputed response functions and reestimated CS+ and CS‐ SEBR amplitudes. We used Experiment 2 to optimize our filter settings and independently validated this on data from Experiment 3 and 4.

### Normalization

Within‐subject normalization of CS+ and CS‐ estimates has been shown to increase predictive validity in SCR analysis, as this reduces the impact of participants with high between‐trials variance at the group level (Staib et al., 2015). In order to test the effect of such normalization, we computed single‐trial estimates of SEBR using our model‐based approach (model M4ST) and all peak‐scoring methods, and *z*‐scored the estimates within each participant, across CS+ and CS‐ trials. We then computed the mean difference CS+/CS‐ from the normalized scores, per participant. Unless otherwise stated, we will discuss results of nonnormalized estimates of the SEBR.

### Model‐Free Methods (Peak‐Scoring)

We compared our model to four existing peak‐scoring methods from the literature, developed and optimized by several research groups. For the first method (we term this B1 from Barker et al., 2014), we band‐pass filtered EMG data with a 4th order Butterworth filter between 28 Hz and 250 Hz, followed by notch filtering to remove 50 Hz harmonics noise (Barker et al., 2014). Rectified signals were smoothed using a 20‐ms moving average. We then computed the maximum startle amplitude between 20 to 120 ms after startle onset and baseline corrected it using the average EMG activity within a 20‐ms time window prior to the onset of the startle stimulus (Barker et al., 2014).

The second peak‐scoring method was adapted from Bradford et al. (2014; termed here Br). EMG data were high‐pass filtered using a 4th order Butterworth filter with cutoff frequency 28 Hz. The filtered signals were rectified and smoothed using another 4th order 30 Hz cutoff Butterworth low‐pass filter. The startle response was quantified as the maximum amplitude between 20 and 120 ms after the startle onset relative to the average from a 50‐ms preonset baseline (Bradford, Kaye, & Curtin, 2014).

As a third peak‐scoring method, we followed the guidelines published by Grillon et al. (1991; termed here G1). We used a 4th order Butterworth filter to band‐pass filter EMG data between 1 Hz and 490 Hz and notch‐filtered to remove 50 Hz harmonics. Filtered data were rectified and smoothed using a 20‐ms moving average. The startle response was quantified as maximum amplitude between 21 and 120 ms after the startle onset relative to the average from a 20‐ms postsound onset baseline.

The fourth peak‐scoring method was adapted from Balderston et al. (2015; termed G2 as it represents a development of algorithm G1). We band‐pass filtered the EMG signal with a 4th order Butterworth filter at 30–490 Hz, and applied a notch filter to remove 50 Hz harmonics. Filtered EMG data were rectified and smoothed using a 20‐ms moving average. The peak startle amplitude for each trial was measured as the maximum EMG amplitude between 20 and 100 ms after startle sound onset (Balderston et al., 2015).

### Methods Comparison

We evaluated the different methods by comparing their ability to predict CS type (CS+/CS‐) from startle amplitude measures on a group level using Bayesian model comparison. For single‐trial methods (M2ST, M4ST, peak‐scoring, SCR), we computed mean CS+ and CS‐ scores per participant; all other methods yield just one value per condition per participant.

To compute model evidence, we used a linear regression model that predicted CS+ and CS‐ type (dependent variable) as linear function of the estimated startle amplitude (independent variable), together with subject‐specific intercepts (across CS type) to account for between‐subjects variability (equivalent to a paired *t* test). The model was inverted using MATLAB's in‐built maximum likelihood function *glmfit*. The residual sum of squares (RSS) from this model was then converted into Akaike information criterion (AIC) by

$$AIC=n*\log\left(\frac{1}{n}RSS\right)+2k,$$

where *n* is the number of observations and *k* is the number of parameters of the predictive model (Burnham & Anderson, 2004). Lower values represent higher model evidence. Note that predictive models from all methods have the same number of parameters. We computed AIC difference between two models as approximation to the relative model evidence for a statement that responses to CS+ and CS‐ are drawn from two distributions with different mean, rather than one distribution. An absolute AIC difference of greater than 3 is usually regarded as decisive. This is because, at a classical α level of α = .05, the probability of the data given the null hypothesis is *p* < .05. Similarly, for an AIC difference larger than 3, the relative probability of the inferior model given the data is *p* < $e^{-3}\;\approx .05$ (Penny, Stephan, Mechelli, & Friston, 2004). In addition to AIC, we also report paired *t* tests and the ensuing Cohen's $d=\frac{t}{\sqrt{n}}$ (where *n* is the sample size) for illustration.

## Results

### Fear Learning During Acquisition

Given that ground truth (i.e., true startle potentiation) is not known, we relied on assuming that participants learned the CS/US association in Experiment 2–4. This assumption was independently confirmed from conditioned SCR during the acquisition phase. In all experiments, anticipatory sympathetic arousal was significantly higher for CS+ than CS‐. This was revealed by paired *t* tests for Experiment 2, *t*(19) = 3.46, *p* < .01, and Experiment 4, *t*(14) = 3.31, *p* < .005. For Experiment 3, a repeated measures analysis of variance (ANOVA) with factors group and CS revealed a significant main effect of CS+/CS‐, *F*(1,28) = 8.02, *p* < .01, but no main effect or interaction involving group.

### Startle Eyeblink Response Function

Using Experiment 1, we extracted epochs of EMG data between 0 to 500 ms with respect to startle sound onset to model our response functions. Figure 1a depicts the mean startle response, across all participants and trials, and the first two PCs. The first and second PC accounted for 59.7% and 9.3% of the total variance across all participants and trials, respectively. Hence, the SEBR can be regarded as rather stereotypical. The second PC resembled a time derivative of the first component. A gamma response function (SEBRF; M1) was fitted on the first component. Model M2 contained the SEBRF together with its time derivative (Figure 1b). Since M1 did not capture the tail of the response sufficiently, we combined the gamma with a Gaussian function (M3; response function not shown in figure). The fitted model parameters are listed in Table 1. The amplitude of the SEBRF, and its derivative in M2, were left as free parameters that were used to calculate the SEBR amplitude corresponding to different conditions. Model M4 was the same as M1 but with latency left as additional free parameter.

**Table 1:** Model Parameters for Initial and Optimized Filter Band

		Model parameters (Before filter optimization)					Model parameters (After filter optimization)		
#	Model description	*k*	θ	*x*_0_	µ	σ	*k*	θ	*x*_0_
M1	SEBRF	2.5320	0.0154	0.0383	–	–	–	–	–
M2ST	SEBRF + SEBRF'	2.5320	0.0154	0.0383	–	–	3.7167	0.0103	0.0340
M3	SEBRF + Gaussian function	2.5320	0.0154	0.0383	0.2119	0.1854	–	–	–
M4ST	SEBRF with latency as free parameter)	2.5320	0.0154	0.0383	–	–	3.5114	0.0108	0.0345

*Note*. Initial filter band 28–250 Hz, optimized filter bands 60–480 Hz (M2ST) and 50–470 Hz (M4ST).

### Initial Model Comparison During Fear Retention

**Figure 2:**
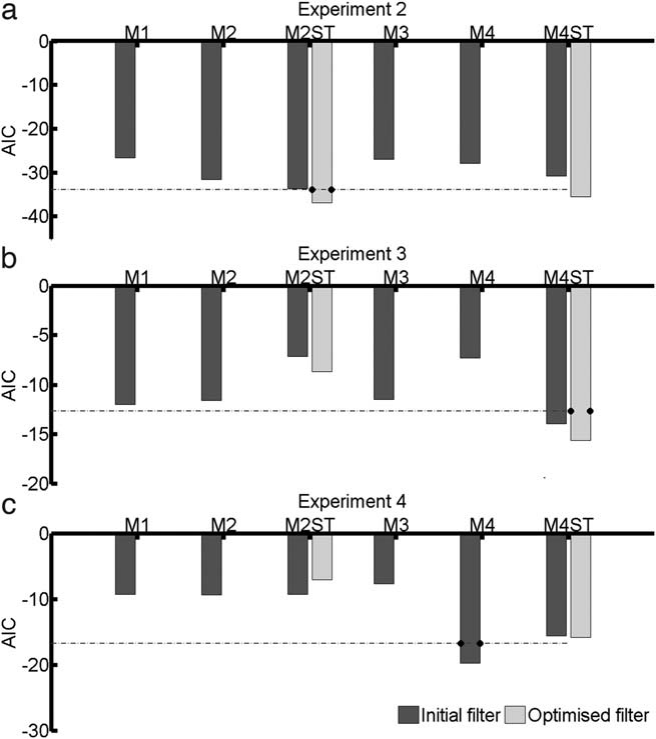
Initial model comparison and filter optimization. The graph shows predictive validity (i.e., ability to distinguish CS+ from CS‐ responses) quantified as Akaike information criterion (AIC, smaller is better). Dark gray: initial filter settings; light gray: optimized filter settings. Dashed lines represent the decision thresholds with respect to the best model (absolute AIC difference > 3). a: Experiment 2. b: Experiment 3. c: Experiment 4.

In Experiment 2, all models were able to distinguish between CS+ and CS‐, while in Experiment 3, all models except M2ST and M4 discriminated between CS+ and CS‐ (Table 2). We used AIC as a measure of predictive validity where smaller AIC reflects better discrimination between CS+ and CS‐. In Experiment 2, the best model M2ST performed marginally but not decisively better than model M4ST (AIC difference = 2.92), such that both models were chosen for further analysis. In Experiment 3, where the startle sound onset was not recorded online, model M4ST performed decisively better than M2ST (Figure 2b; black bars), possibly due to small variation in startle sound onset not captured in the GLM inversion of models M1–M3.

**Table 2:** Paired T Test for the Difference Between CS+/CS‐ for Different Models with Initial Filter Band (28–250 Hz)

		Experiment 2			Experiment 3			Experiment 4		
#	Model description	*t*(19)	*p*	Cohen's *d*	*t*(14)	*p*	Cohen's *d*	*t*(14)	*p*	Cohen's *d*
M1	SEBRF	4.23	<.001	0.95	2.62	<.05	0.68	2.26	<.05	0.58
M2	SEBRF + SEBRF'	4.78	<.001	1.07	2.57	<.05	0.67	2.26	<.05	0.58
M2ST	SEBRF + SEBRF' (single trial)	5.00	<.0001	1.12	1.94	.07	0.50	2.26	<.05	0.58
M3	SEBRF + Gaussian function	4.27	<.001	0.95	2.55	<.05	0.66	2.02	.06	0.52
M4	SEBRF with latency as free parameter	4.37	<.001	0.98	1.97	.07	0.51	3.61	<.005	0.93
M4ST	SEBRF with latency as free parameter (single trial)	4.68	<.001	1.05	2.89	<.05	0.74	3.08	<.01	0.80

*Note*. See corresponding Figure 2 for AIC.

### Filter Optimization

Next, we searched for filter settings that maximized predictive validity of model M2ST and M4ST for Experiment 2. For each filter setting, the response function was refitted. We varied the high cutoff frequency between 10–90 Hz and the low cutoff frequency between 20–490 Hz. Changing the high‐pass cutoff frequency had little impact on the model performance. A band‐pass filter with cutoff frequencies of 60–480 Hz resulted in the best predictive validity for model M2ST while the best filter band for M4ST was 50–470 Hz. The fitted model parameters are listed in Table 1.

Predictive validity for model M2ST with optimized filter settings for Experiment 2 was decisively improved when compared to our initial filter settings (AIC difference: 3.24, Figure 2a). Similarly, model M4ST with optimal filter settings discriminated CS+ and CS‐ decisively better than initial model M4ST (AIC difference 4.79; Figure 2a). Crucially, M2ST and M4ST with optimized filters did not decisively differ from each other (AIC difference 1.37). Next, we validated our optimal models M2ST and M4ST in fear retention Experiment 3. As in the initial comparison, M4ST performed decisively better than all other models (Figure 2b; light gray bars). Based on its superiority in Experiment 3 and its noninferiority in Experiment 2, we chose model M4ST as the final model with an optimized filter band of 50–470 Hz.

### Model Comparison During Fear Acquisition

To validate the generalizability of our model, we computed the predictive validity of SEBR to distinguish CS+/CS‐ during fear acquisition in Experiment 4. We show performance of all our models with initial filter settings along with optimal filters (Figure 2c; black bars—initial filter settings, and gray bars—optimized filter settings). M4ST again performed decisively better than model M2ST and all other models except model M4, which was decisively better than all other model‐based methods.

### Comparison with Peak‐Scoring Methods

**Figure 3:**
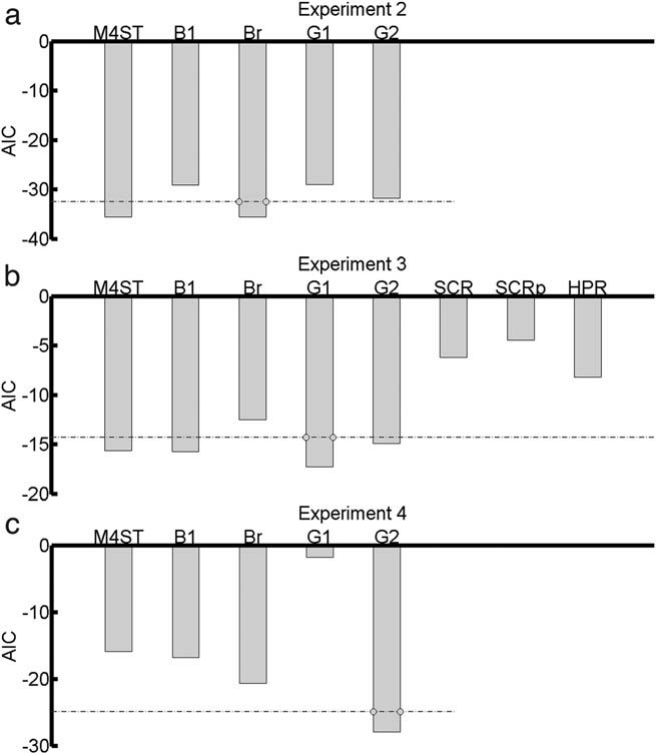
Comparison of M4ST with other methods. The graph shows predictive validity, quantified as Akaike information criterion (AIC, smaller is better) for model M4ST, four peak‐scoring methods (B1, Br, G1, G2), SCR (model‐based analysis), peak‐scored SCR (SCRp), and HPR. Dashed lines represent the decision thresholds with respect to the best model (absolute AIC difference > 3). a: Experiment 2. b: Experiment 3. c: Experiment 4.

Next, we compared predictive validity of M4ST with four different peak‐scoring methods (Table 3, Figure 3). In Experiment 2, our model performed similar to peak‐scoring method Br and decisively better than the other three peak‐scoring methods (Figure 3a). As filter settings in our model were optimized on Experiment 2, the comparison of optimized model‐based analysis and peak‐scoring methods might be biased. In Experiment 3, M4ST performed decisively better than peak‐scoring method Br and similar to all other peak‐scoring methods (Figure 3b). However, in Experiment 4, M4ST was decisively less sensitive than peak‐scoring methods G2 and Br, comparable to B1, and decisively better than G1 (Figure 3c).

**Table 3:** Paired T Test for the Difference Between CS+/CS‐ for Different Methods

		Experiment 2			Experiment 3			Experiment 4		
#	Model description	*t*(19)	*p*	Cohen's *d*	*t*(14)	*p*	Cohen's *d*	*t*(14)	*p*	Cohen's *d*
M4ST	Best model‐based method	5.21	<.0001	1.17	3.09	<.05	0.80	3.12	<.01	0.81
B1	Barker et al. (2014)	4.51	<.001	1.01	3.10	<.05	0.80	3.23	<.01	0.96
Br	Bradford et al. (2014)	5.21	<.0001	1.17	2.69	<.05	0.69	3.72	<.005	0.96
G1	Grillon et al. (1991)	4.49	<.001	1.00	3.30	<.01	0.85	0.92	.37	0.24
G2	Balderston et al. (2015)	4.80	<.001	1.07	3.00	<.05	0.77	4.63	<.001	1.20
SCR	Model‐based SCR analysis (Staib et al., 2015)				1.79	.10	0.46			
SCR_p	Peak‐scoring SCR analysis (Boucsein, 2012)				1.49	.16	0.39			
HPR	Model‐based HPR analysis (Castegnetti et al., 2016)				2.10	.06	0.54			

*Note*. See corresponding Figure 3 for AIC. M4ST uses optimized filter band (50–470 Hz).

### Effect of Normalization

**Figure 4:**
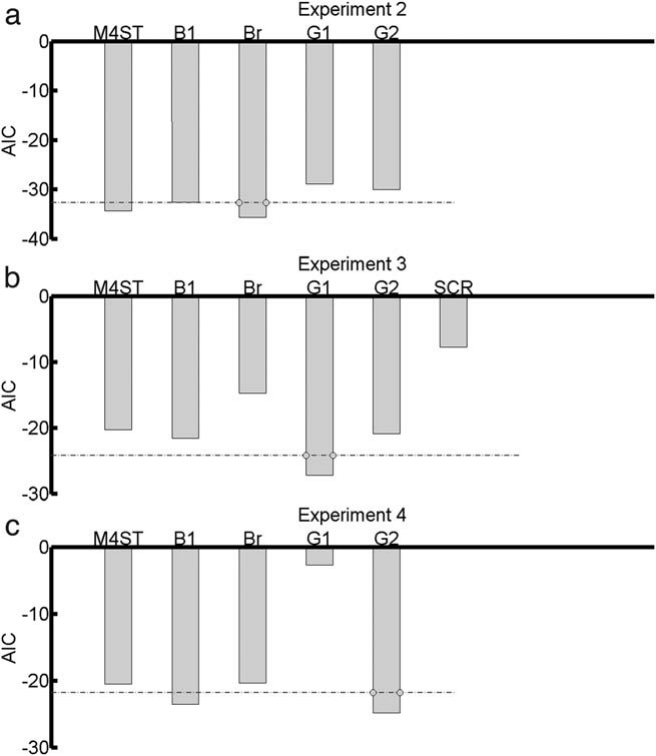
Comparison of M4ST with other methods for trial‐by‐trial normalized response measures. The graph shows predictive validity, quantified as Akaike information criterion (AIC, smaller is better) for model M4ST, four peak‐scoring methods (B1, Br, G1, G2), and SCR. Dashed lines represent the decision thresholds with respect to the best model (absolute AIC difference > 3). a: Experiment 2. b: Experiment 3. c: Experiment 4.

We sought to investigate the effect of normalizing single‐trial SEBR estimates on predictive validity, both our model‐based (M4ST) and all the peak‐scoring methods (Figure 4, Table 4). Predictive validity for Experiment 2 did not decisively differ for nonnormalized or normalized estimates obtained from model M4ST (AIC difference 1.18). Normalizing the estimates of peak‐scoring methods resulted in a decisively higher predictive validity for method B1 (AIC difference 3.45) but not for the remaining methods. In Experiment 3, normalizing resulted in an improved performance for M4ST (AIC difference 4.61) as well as for methods B1, G1, and G2 (AIC difference 5.90, 9.90, and 6.00, respectively) but not for Br. Similarly in Experiment 4, normalizing the estimates resulted in significantly better discriminatory power for model M4ST (AIC difference 4.64) and peak scoring method B1 and G2 (AIC difference 7.04) but not Br and G1, while G2 became significantly worse (AIC difference 3.14).

**Table 4:** Paired T Test for the Difference Between CS+/CS‐ for Normalized Estimates from Different Methods

		Experiment 2			Experiment 3			Experiment 4		
#	Model description	*t*(19)	*p*	Cohen's *d*	*t*(14)	*p*	Cohen's *d*	*t*(14)	*p*	Cohen's *d*
M4ST	Best model‐based method	5.08	<.0001	1.14	3.67	<.01	0.95	3.70	<.005	0.96
B1	Barker et al. (2014)	4.88	<.001	1.09	3.84	<.01	0.99	4.12	<.005	1.06
Br	Bradford et al. (2014)	5.23	<.0001	1.17	2.98	<.05	0.77	3.68	<.005	0.95
G1	Grillon et al. (1991)	4.48	<.001	1.00	4.54	<.001	1.17	1.17	.26	0.30
G2	Balderston et al. (2015)	4.61	<.001	1.03	3.75	<.01	0.97	4.24	<.001	1.10
SCR	Model‐based SCR analysis (Staib et al., 2015)				2.03	.06	0.52			

*Note*. See corresponding Figure 4 for AIC. M4ST uses optimized filter band (50–470 Hz).

### Comparison to SCR and HPR

We sought to put our results and, in particular, the differences between different SEBR analysis methods into the context of other psychophysiological measures. To this end, half of the participants in Experiment 3 were not exposed to startle sounds during the retention test (no‐startle group) such that we could analyze their SCR and HPR. Results are included in Table 3 and 4 and show that SEBR is by far more sensitive than measures derived from SCR and HPR.

### SEBR as a Function of Number of Extinction Trials

**Figure 5:**
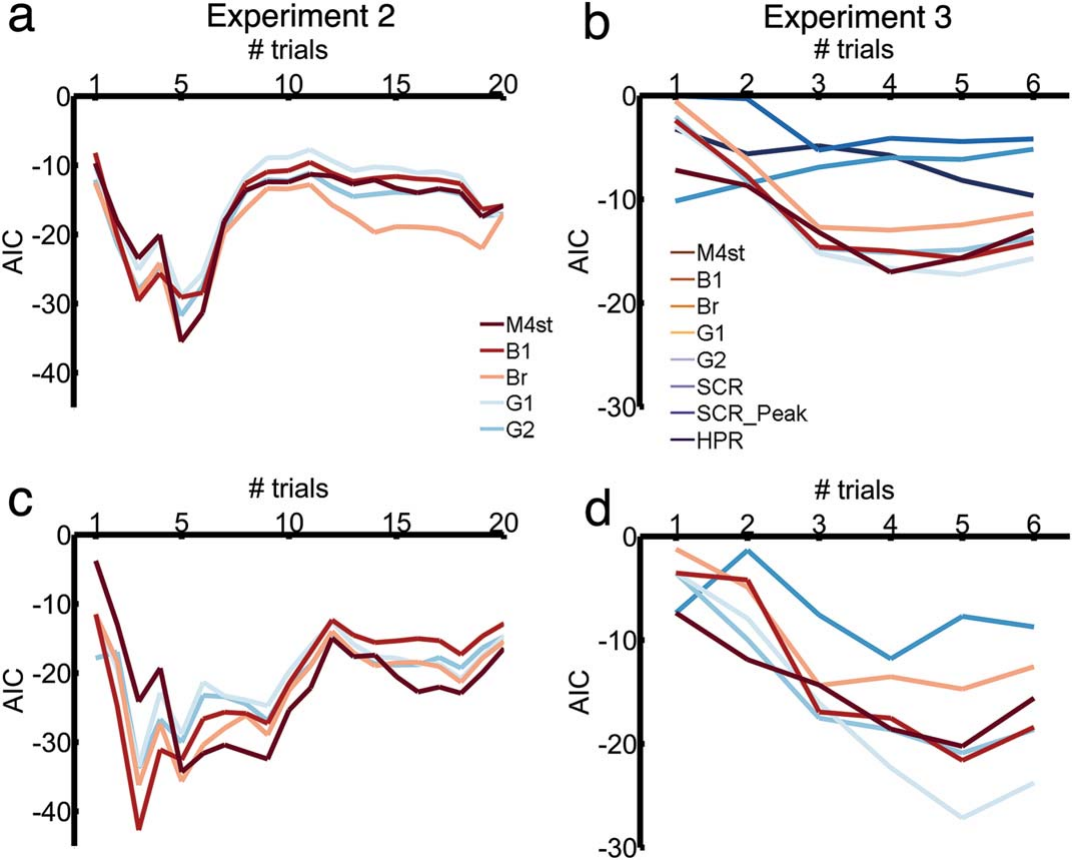
Comparison of different number of trials included in analysis, for model M4ST, four peak‐scoring methods (B1, Br, G1, G2), SCR (model‐based analysis), peak‐scored SCR (SCR_Peak), and HPR. The graph shows predictive validity, quantified as Akaike information criterion (AIC, smaller is better). a: Experiment 2, nonnormalized response measures. b: Experiment 2, nonnormalized response measures. c: Experiment 2, normalized response measures. d: Experiment 3, normalized response measures.

When measuring fear retention under extinction, a critical question is how many trials to include in the analysis: increasing trial number may increase the signal‐to‐noise ratio during measurement, but also include trials with weakened fear memory. To investigate how fear memory can be assessed in the retention session, we computed the predictive validity of each modality and method as a function of the number of trials included. For Experiment 2, the highest predictive validity was obtained for three CS+ and three CS‐ trials, with method B1 (AIC −42.72, Figure 5) and normalized estimates. This was followed by method Br (AIC −36.21 for three trials and normalized estimates and −35.51 for five trials and nonnormalized estimates), and by M4ST (AIC −35.49 for five trials and nonnormalized estimates and −34.48 for five trials and normalized estimates).

For Experiment 3, the highest predictive validity was observed for method G1 (AIC −27.16), when considering the first five trials of each CS+/CS‐ normalized responses. This was followed by methods B1 and G2 (AIC −21.60 and −20.88, respectively) and model‐based method M4ST, with an AIC of −20.24, for five trials and normalized estimates. All startle approaches outperformed the other two modalities. SCR had the best predictive validity for four trials (AIC −11.8, for normalized estimates) and HPR for six trials (AIC −9.65). The peak‐scoring approach for SCR gave the highest predictive validity when considering three trials (AIC of −5.27), which was decisively worse than the one obtained for model‐based SCR analysis.

## Discussion

In this study, we developed a novel model‐based analysis method for SEBR and validated its suitability for quantifying fear learning in humans. We first derived a canonical SEBRF to model orbicularis oculi EMG responses to a startle probe. From a range of possible models, model M4ST emerged as the most robust model in fear retention Experiment 2–3. This model explicitly estimates SEBR latency and amplitude on individual trials using a dictionary‐matching algorithm, and can thus account for small variations in startle latency. We show that this model also generalizes to a different data set during fear acquisition (Experiment 4). Analysis of four extant peak‐scoring methods (Balderston et al., 2015; Barker et al., 2014; Bradford et al., 2014; Grillon et al., 1991) yields a heterogeneous picture. For each data set, a different peak‐scoring method emerges as winning method. Crucially, the winning method from each experiment is significantly outperformed by another peak‐scoring method in another experiment. This is even the case when comparing the two very similar fear retention Experiments 2–3, while M4ST is among the winning methods for both data sets. Thus, the model‐based approach appears to be a robust method that generalizes to different experimental circumstances. However, the observed heterogeneity in peak‐scoring methods urges further investigation. Finally, we observed that within‐subject normalization of CS+/CS‐ estimates resulted in higher predictive validity for most, but not all methods.

All startle analysis methods significantly outperformed SCR/HPR. While SEBR and SCR are common measures to quantify fear, HPR is less often used. A possible reason is that, due to respiratory arrhythmia, HPR is a relatively noisy measure, and peak‐scoring HPR requires averaging over many trials. This is different from SCR or SEBR, which can be scored on a single‐trial level. Overall, this comparison suggests that SEBR can assess fear retention more precisely than SCR or HPR.

From our findings, we conclude that our model provides a powerful method to infer human fear learning from SEBR. However, several model limitations need to be taken into account. We assumed that the SEBR can be described by a LTI system and, thus, that the response to two subsequent stimuli can be represented by pure summation of their independent output. This assumption is obviously irrelevant for experiments with an interval between startle probes that exceeds the duration of the SEBR, which is typically the case in fear‐potentiated startle studies. Also, in affective startle modulation paradigms, in which startle magnitude is smallest during pleasant emotional state and largest during unpleasant state (Cuthbert, Bradley, & Lang, 1996), long ITIs are typically used. Thus, our model could be applied well in these experimental paradigms to differentiate between pleasant and unpleasant stimuli. In contrast, paradigms to investigate prepulse inhibition of the acoustic startle response employ a brief nonstartling stimulus before the startle probe (Wynn et al., 2004). Because the prepulse can by itself elicit a startle response (Blumenthal et al., 2005), the LTI assumption may not be appropriate for this situation. Moreover, the prepulse may potentially change the shape of the SEBR. However, these issues are, in principle, accessible with the modeling technique used here, and the applicability of the current model to prepulse inhibition experiments could be assessed in further experiments. Additionally, differences in laboratory settings and equipment may result in variations in the shape of the startle response, and this mandates our model to be tested under different conditions and experimental paradigms.

While our model well captures SEBR amplitude differences between CS+ and CS‐, the ultimate goal is to also measure small changes in fear potentiation. It is a challenging question whether any SEBR analysis method can capture small differences in CS+ reinforcement probability, or US magnitude. Crucially, startle amplitude seems to change with US probability or strength, but the relation between SEBR and expected US magnitude is not necessarily linear (Bach, 2015), and, under some circumstances, it is even nonmonotonic (Davis & Astrachan, 1978). Thus, at present our model (or any peak‐scoring approach) does not allow inferring predicted US magnitude or probability from measured SEBR. Deriving such a relation remains a task for future investigations.

To summarize, our model is consistently able to differentiate CS+ from CS‐ responses in different experimental setups, during fear extinction and acquisition, and can be readily used for measuring fear retention. By contrast, peak‐scoring methods show a large heterogeneity across data sets and may thus generalize less well to different experimental circumstances. Thus, the model‐based approach shows more versatility compared to other investigated methods. With this work, we hope to contribute to ongoing research efforts aimed at maximizing the sensitivity of fear memory measures.

## References

[psyp12775-bib-0001] Ameli, R. , Ip, C. , & Grillon, C. (2001). Contextual fear‐potentiated startle conditioning in humans: Replication and extension. Psychophysiology, 38(3), 383–390. doi: 10.1111/1469-8986.3830383 11352126

[psyp12775-bib-0002] Andreatta, M. , & Pauli, P. (2015). Appetitive vs. aversive conditioning in humans. Frontiers in Behavioral Neuroscience, 9, 128. doi: 10.3389/fnbeh.2015.00128 26042011 10.3389/fnbeh.2015.00128 PMC4436895

[psyp12775-bib-0003] Bach, D. R. (2014). A head‐to‐head comparison of SCRalyze and Ledalab, two model‐based methods for skin conductance analysis. Biological Psychology, 103(1), 63–68. doi: 10.1016/j.biopsycho.2014.08.006 25148785 10.1016/j.biopsycho.2014.08.006 PMC4266536

[psyp12775-bib-0004] Bach, D. R. (2015). A cost minimisation and Bayesian inference model predicts startle reflex modulation across species. Journal of Theoretical Biology, 370(0), 53–60. doi: 10.1016/j.jtbi.2015.01.031 25660056 10.1016/j.jtbi.2015.01.031 PMC4371795

[psyp12775-bib-0005] Bach, D. R. , Daunizeau, J. , Friston, K. J. , & Dolan, R. J. (2010). Dynamic causal modelling of anticipatory skin conductance responses. Biological Psychology, 85(1), 163–170. doi: 10.1016/j.biopsycho.2010.06.007 20599582 10.1016/j.biopsycho.2010.06.007 PMC2923733

[psyp12775-bib-0006] Bach, D. R. , Flandin, G. , Friston, K. J. , & Dolan, R. J. (2009). Time‐series analysis for rapid event‐related skin conductance responses. Journal of Neuroscience Methods, 184(2), 224–234. doi: 10.1016/j.jneumeth.2009.08.005 19686778 10.1016/j.jneumeth.2009.08.005 PMC2772899

[psyp12775-bib-0007] Bach, D. R. , & Friston, K. J. (2013). Model‐based analysis of skin conductance responses: Towards causal models in psychophysiology. Psychophysiology, 50(1), 15–22. doi: 10.1111/j.1469-8986.2012.01483.x 23094650 10.1111/j.1469-8986.2012.01483.x

[psyp12775-bib-0008] Bach, D. R. , Friston, K. J. , & Dolan, R. J. (2013). An improved algorithm for model‐based analysis of evoked skin conductance responses. Biological Psychology, 94(3), 490–497. doi: 10.1016/j.biopsycho.2013.09.010 24063955 10.1016/j.biopsycho.2013.09.010 PMC3853620

[psyp12775-bib-0009] Bach, D. R. , Gerster, S. , Tzovara, A. , & Castegnetti, G. (2016). A linear model for event‐related respiration responses. Journal of Neuroscience Methods, 270, 147–155. doi: 10.1016/j.jneumeth.2016.06.001 27268156 10.1016/j.jneumeth.2016.06.001 PMC4994768

[psyp12775-bib-0010] Balderston, N. L. , Mathur, A. , Adu‐Brimpong, J. , Hale, E. A. , Ernst, M. , & Grillon, C. (2015). Effect of anxiety on behavioural pattern separation in humans. Cognition and Emotion. Advance online publication. doi: 10.1080/02699931.2015.1096235 10.1080/02699931.2015.1096235 PMC4993678 26480349

[psyp12775-bib-0011] Barker, T. V. , Reeb‐Sutherland, B. C. , & Fox, N. A. (2014). Individual differences in fear potentiated startle in behaviorally inhibited children. Developmental Psychobiology, 56(1), 133–141. doi: 10.1002/dev.21096 23341151 10.1002/dev.21096 PMC4123448

[psyp12775-bib-0012] Blumenthal, T. D. (1988). The startle response to acoustic stimuli near startle threshold: Effects of stimulus rise and fall time, duration, and intensity. Psychophysiology, 25 *(* 5 *)*, 607–611. doi: 10.1111/j.1469-8986.1988.tb01897.x 3186888 10.1111/j.1469-8986.1988.tb01897.x

[psyp12775-bib-0013] Blumenthal, T. D. , Cuthbert, B. N. , Filion, D. L. , Hackley, S. , Lipp, O. V. , & Van Boxtel, A. (2005). Committee report: Guidelines for human startle eyeblink electromyographic studies. Psychophysiology, 42(1), 1–15. doi: 10.1111/j.1469-8986.2005.00271.x 15720576 10.1111/j.1469-8986.2005.00271.x

[psyp12775-bib-0014] Boucsein, W. (2012). Electrodermal activity. New York, NY: Springer. doi: 10.1007/978-1-4614-1126-0

[psyp12775-bib-0015] Bradford, D. E. , Kaye, J. T. , & Curtin, J. J. (2014). Not just noise: Individual differences in general startle reactivity predict startle response to uncertain and certain threat. Psychophysiology, 51(5), 407–411. doi: 10.1111/psyp.12193 24611542 10.1111/psyp.12193 PMC3984356

[psyp12775-bib-0016] Brown, J. S. , Kalish, H. I. , & Farber, I. E. (1951). Conditioned fear as revealed by magnitude of startle response to an auditory stimulus. Journal of Experimental Psychology, 41(5), 317–328. doi: 10.1037/h0060166 14861383 10.1037/h0060166

[psyp12775-bib-0017] Brown, L. A. , LeBeau, R. T. , Chat, K. Y. , & Craske, M. G. (2016). Associative learning versus fear habituation as predictors of long‐term extinction retention. Cognition and Emotion, 9931, 0–12. doi: 10.1080/02699931.2016.1158695 10.1080/02699931.2016.1158695 26998883

[psyp12775-bib-0018] Burnham, K. P. , & Anderson, D. R. (2004). Multimodel inference\understanding AIC and BIC in model selection. Sociological Methods & Research, 33, 261–304.

[psyp12775-bib-0019] Carew, T. J. , Walters, E. T. , & Kandel, E. R. (1981). Classical conditioning in a simple withdrawal reflex in Aplysia californica. Journal of Neuroscience, 1(12), 1426–1437. doi: 10.1177/0269881107067097 7320755 10.1523/JNEUROSCI.01-12-01426.1981 PMC6564124

[psyp12775-bib-0020] Carmichael, O. , & Lockhart, S. (2012). The role of diffusion tensor imaging in the study of cognitive aging. Brain Imaging in Behavioral Neuroscience *, (November*), 289–320. doi: 10.1007/7854 10.1007/7854_2011_176 22081443

[psyp12775-bib-0021] Castegnetti, G. , Tzovara, A. , Staib, M. , Paulus, P. C. , Hofer, N. , & Bach, D. R. (2016). Modeling fear‐conditioned bradycardia in humans. Psychophysiology, 53, 930–939. doi:10.1111/psyp.12637 26950648 10.1111/psyp.12637 PMC4869680

[psyp12775-bib-0022] Cuthbert, B. N. , Bradley, M. M. , & Lang, P. J. (1996). Probing picture perception: Activation and emotion. Psychophysiology, 33(2), 103–111. doi: 10.1111/j.1469-8986.1996.tb02114.x 8851238 10.1111/j.1469-8986.1996.tb02114.x

[psyp12775-bib-0023] Davis, M. , & Astrachan, D. I. (1978). Conditioned fear and startle magnitude: Effects of different footshock or backshock intensities used in training. Journal of Experimental Psychology. Animal Behavior Processes, 4(2), 95–103. doi: 10.1037/0097-7403.4.2.95 670892 10.1037//0097-7403.4.2.95

[psyp12775-bib-0024] Friston, K. J. , Fletcher, P. , Josephs, O. , Holmes, A. , Rugg, M. D. , & Turner, R. (1998). Event‐related fMRI: characterizing differential responses. NeuroImage, 7(1), 30–40. doi: 10.1006/nimg.1997.0306 9500830 10.1006/nimg.1997.0306

[psyp12775-bib-0025] Grillon, C. , Ameli, R. , Woods, S. W. , Merikangas, K. , & Davis, M. (1991). Fear‐potentiated startle in humans: Effects of anticipatory anxiety on the acoustic blink reflex. Psychophysiology, 28(5), 588–595. doi: 10.1111/j.1469-8986.1991.tb01999.x 1758934 10.1111/j.1469-8986.1991.tb01999.x

[psyp12775-bib-0026] Grillon, C. , & Baas, J. (2003). A review of the modulation of the startle reflex by affective states and its application in psychiatry. Clinical Neurophysiology, 114(9), 1557–1579. doi: 10.1016/S1388-2457(03)00202-5 12948786 10.1016/s1388-2457(03)00202-5

[psyp12775-bib-0027] Grillon, C. , Cordova, J. , Morgan, C. A. , Charney, D. S. , & Davis, M. (2004). Effects of the beta‐blocker propranolol on cued and contextual fear conditioning in humans. Psychopharmacology, 175(3), 342–352. doi: 10.1007/s00213-004-1819-5 15007536 10.1007/s00213-004-1819-5

[psyp12775-bib-0028] Hamm, A. O. , & Vaitl, D. (1996). Affective learning: Awareness and aversion. Psychophysiology, 33(6), 698–710. doi: 10.1111/j.1469-8986.1996.tb02366.x 8961792 10.1111/j.1469-8986.1996.tb02366.x

[psyp12775-bib-0029] Hygge, S. , & Hugdahl, K. (1985). Skin conductance recordings and the NaCl concentration of the electrolyte. Psychophysiology, 22(3) 365–367. 4011809 10.1111/j.1469-8986.1985.tb01616.x

[psyp12775-bib-0030] Kluge, C. , Bauer, M. , Leff, A. P. , Heinze, H.‐J. , Dolan, R. J. , & Driver, J. (2011). Plasticity of human auditory‐evoked fields induced by shock conditioning and contingency reversal. Proceedings of the National Academy of Sciences of the United States of America, 108(30), 12545–12550. doi: 10.1073/pnas.1016124108 21746922 10.1073/pnas.1016124108 PMC3145740

[psyp12775-bib-0031] Korn, C. W. , & Bach, D. R. (2016). A solid frame for the window on cognition: Modeling event‐related pupil responses. Journal of Vision, 16, 28. 10.1167/16.3.28 PMC4993241 26894512

[psyp12775-bib-0032] Korn, C. W. , Staib, M. , Tzovara, A. , Castegnetti, G. , & Bach, D. R. (2016). A pupil size response model to assess fear learning. Psychophysiology. Manuscript in preparation. 10.1111/psyp.12801 PMC5324687 27925650

[psyp12775-bib-0033] Lagarias, J. C. , Reeds, J. A. , Wright, M. H. , & Wright, P. E. (1998). Convergence properties of the Nelder–Mead simplex method in low dimensions. SIAM Journal on Optimization, 9(1), 112–147. doi: 10.1137/S1052623496303470

[psyp12775-bib-0034] Lang, P. J. , Bradley, M. M. , & Cuthbert, B. N. (1997). Motivated attention: Affect, activation, and action In Lang P. J., Simons R. F., & Balaban M. (Eds.), Attention and orienting: Sensory and motivational processes (pp. 97–135). Mahwah, NJ: Lawrence Erlbaum.

[psyp12775-bib-0035] LeDoux, J. E. (1998). The emotional brain: The mysterious underpinnings of emotional life. New York, NY: Simons & Schuster. doi: 10.2307/3953278

[psyp12775-bib-0036] Lindner, K. , Neubert, J. , Pfannmöller, J. , Lotze, M. , Hamm, A. O. , & Wendt, J. (2015). Fear‐potentiated startle processing in humans: Parallel fMRI and orbicularis EMG assessment during cue conditioning and extinction. International Journal of Psychophysiology, 98(3), 535–545. doi: 10.1016/j.ijpsycho.2015.02.025 25725377 10.1016/j.ijpsycho.2015.02.025

[psyp12775-bib-0037] Lipp, O. V , Sheridan, J. , & Siddle, D. A. (1994). Human blink startle during aversive and nonaversive Pavlovian conditioning. Journal of Experimental Psychology. Animal Behavior Processes, 20(4), 380–389. doi: 10.1037//0097-7403.20.4.380 7964520

[psyp12775-bib-0038] Lissek, S. , Powers, A. S. , McClure, E. B. , Phelps, E. A , Woldehawariat, G. , Grillon, C. , & Pine, D. S. (2005). Classical fear conditioning in the anxiety disorders: A meta‐analysis. Behaviour Research and Therapy, 43(11), 1391–424. doi: 10.1016/j.brat.2004.10.007 15885654 10.1016/j.brat.2004.10.007

[psyp12775-bib-0039] Pan, J. , & Tompkins, W. J. (1985). A real‐time QRS detection algorithm. IEEE Transactions on Biomedical Engineering, 3, 23–236. doi: 10.1109/TBME.1985.325532 10.1109/TBME.1985.325532 3997178

[psyp12775-bib-0040] Paulus, P. C. , Castegnetti, G. , & Bach, D. R. (2016). Modeling event‐related heart period responses. Psychophysiology, 53, 837–846. doi: 10.1111/psyp.12622 26849101 10.1111/psyp.12622 PMC4869677

[psyp12775-bib-0041] Penny, W. D. , Stephan, K. E. , Mechelli, A , & Friston, K. J. (2004). Comparing dynamic causal models. NeuroImage, 22(3), 1157–1172. doi: 10.1016/j.neuroimage.2004.03.026 15219588 10.1016/j.neuroimage.2004.03.026

[psyp12775-bib-0042] Reinhard, G. , Lachnit, H. , & König, S. (2006). Tracking stimulus processing in Pavlovian pupillary conditioning. Psychophysiology, 43(1), 73–83. doi: 10.1111/j.1469-8986.2006.00374.x 16629687 10.1111/j.1469-8986.2006.00374.x

[psyp12775-bib-0043] Reist, C. , Duffy, J. G. , Fujimoto, K. , & Cahill, L. (2001). β‐Adrenergic blockade and emotional memory in PTSD. International Journal of Neuropsychopharmacology, 4(4), 377–383. 11806863 10.1017/S1461145701002607

[psyp12775-bib-0044] Schiller, D. , Monfils, M.‐H. , Raio, C. M. , Johnson, D. C. , Ledoux, J. E. , & Phelps, E. A. (2010). Preventing the return of fear in humans using reconsolidation update mechanisms. Nature, 463(7277), 49–53. doi: 10.1038/nature08637 20010606 10.1038/nature08637 PMC3640262

[psyp12775-bib-0045] Staib, M. , Castegnetti, G. , & Bach, D. R. (2015). Optimising a model‐based approach to inferring fear learning from skin conductance responses. Journal of Neuroscience Methods, 255, 131–138. doi: 10.1016/j.jneumeth.2015.08.009 26291885 10.1016/j.jneumeth.2015.08.009 PMC4612446

[psyp12775-bib-0046] VanElzakker, M. B. , Dahlgren, K. M. , Davis, C. F. , Dubois, S. , & Shin, L. M. (2014). From Pavlov to PTSD: The extinction of conditioned fear in rodents, humans, and anxiety disorders. Neurobiology of Learning and Memory, 113, 3–18. doi: 10.1016/j.nlm.2013.11.014 24321650 10.1016/j.nlm.2013.11.014 PMC4156287

[psyp12775-bib-0047] Vanman, E. J. , Boehmelt, A. H. , Dawson, M. E. , & Schell, A. M. (1996). The varying time courses of attentional and affective modulation of the startle eyeblink reflex. Psychophysiology, 33(6) 691–697. doi: 10.1111/j.1469-8986.1996.tb02365.x 8961791 10.1111/j.1469-8986.1996.tb02365.x

[psyp12775-bib-0048] Wynn, J. K. , Dawson, M. E. , Schell, A. M. , McGee, M. , Salveson, D. , & Green, M. F. (2004). Prepulse facilitation and prepulse inhibition in schizophrenia patients and their unaffected siblings. Biological Psychiatry, 55(5), 518–523. doi: 10.1016/j.biopsych.2003.10.018 15023580 10.1016/j.biopsych.2003.10.018

[psyp12775-bib-0049] Yeomans, J. S. , Li, L. , Scott, B. W. , & Frankland, P. W. (2002). Tactile, acoustic and vestibular systems sum to elicit the startle reflex. Neuroscience and Biobehavioral Reviews, 26(1), 1–11. doi: 10.1016/S0149-7634(01)00057-4 11835980 10.1016/s0149-7634(01)00057-4

